# Influence of Rubber Size on Properties of Crumb Rubber Mortars

**DOI:** 10.3390/ma9070527

**Published:** 2016-06-29

**Authors:** Yong Yu, Han Zhu

**Affiliations:** 1 Department of Civil Engineering, Tianjin University, Tianjin 300350, China; shourimojie@tju.edu.cn; 2 Key Laboratory of Coast Civil Structure Safety, Tianjin University, Ministry of Education, Tianjin 300072, China

**Keywords:** rubber particle size, crumb rubber mortar, pore structure, mechanical properties, drying shrinkage

## Abstract

Studies on the properties and applications of rubber cement-based materials are well documented. The sizes of rubbers used in these materials varied. However, information about the effects of rubber size on the properties of rubber cement-based materials, especially pore structure, mechanical strengths, and drying shrinkage properties, remains limited. Three groups of rubber with major particle sizes of 2–4 mm, 1–3 mm, and 0–2 mm were selected in this study. This paper presents experimental studies on the effects of rubber size on the consistency, fresh density, pore structure, mechanical properties, and drying shrinkage properties of crumb rubber mortars (CRMs). Results demonstrated that the consistency and fresh density of CRMs decreased with the rubber size. As to the pore structure, the total pore volume increased with the decrease of the rubber size. By contrast, the influence of the rubber size on the mesopore (<50 nm) volume is not as significant as that of the rubber content. The mechanical properties of CRMs decreased with the rubber size. Low rubber stiffness and large pore volumes, especially those of small sized rubbers, contribute to the reduction of CRMs strength. The drying shrinkage of CRM increases as the rubber size decreases. The influences of rubber size on capillary tension are not significant. Thus, the shrinkage increases with the decrease of rubber size mainly because of its function in the deformation modulus reduction of CRMs.

## 1. Introduction

The reduction of energy consumption and carbon emissions has become a global movement [1]. In recent years, irrespective of political, economic, or ecological reasons, recycling has been encouraged worldwide [2]. The use of waste tires as cement-based material additives is a possible disposal solution because waste tire management is increasingly becoming a significant environmental, health, and aesthetic problem that is difficult to solve [3]. A major advantage of incorporating tire rubber wastes to cement materials is increased ductility and flexibility [4,5], although compressive strength is reduced [6,7]; tire rubber is widely introduced in engineering structures. Studies introduced rubber concrete into rigid pavements [8], airport pavement [9], and steel–concrete composite beams [10,11]. The possibility of using rubber concrete as a structural material to enhance the dynamic performance and reduce seismic response of concrete structures has also been studied [12].

With the development of rubber concrete applications, a series of studies has been conducted by scholars worldwide. However, some results on the influences of rubber particles on cement-based materials are inconsistent. Studies [13,14] showed that recycled rubber-filled cement-based materials achieved workability that is comparable or better than the control cement-based materials. However, research [15] showed that the workability of cement mortar decreased as the crumb rubber content increased. Two studies [16,17] showed that the addition of coarse rubber chips in cement-based materials reduced the compressive strength more than the addition of fine crumb rubber did, but another study [18] indicated the opposite trend [19]. Direct comparison of the research results is difficult because the different findings may be caused by the various materials and test methods used. The inconsistent results may also be caused by the different rubber sizes used. Concerning this issue, scholars had studied the influences of rubber size on cement-based materials, and the studies mainly focus on the workability, mechanical strengths, water absorption, resistance to sulphuric acid attack, and carbonation of cement-based materials [20,21,22,23,24]. Other studies [25,26] showed that the rubber particles in the mixture increased porosity. Some reports [27,28,29,30] revealed that the drying shrinkage of rubberized cement-based materials increased with the rubber content. However, our understanding of the effects of crumb rubber size on the porosity structure, mechanical strength, and long-term drying shrinkage properties of CRM remains limited. Both mechanical strength and drying shrinkage are important properties of cement-based materials, and are related to porosity structures. However, when discussing the influences of rubber on CRM, most studies did not take into account the effects of extra porosity caused by rubber.

The present study aims to further understand the properties of cement-based materials made with rubber of varying sizes. Three groups of rubbers were used in this study. A series of tests, including tests on the consistency and density of fresh mortars, pore structures of hardened mortars, mechanical properties, and drying shrinkage of mortars, were conducted in accordance with relevant standards. Influences of rubber size on mechanical strength and drying shrinkage properties of CRM were discussed considering the function of rubber on pore structures.

## 2. Experimental Program

### 2.1. Materials and Mixture Proportions

**Figure 1:**
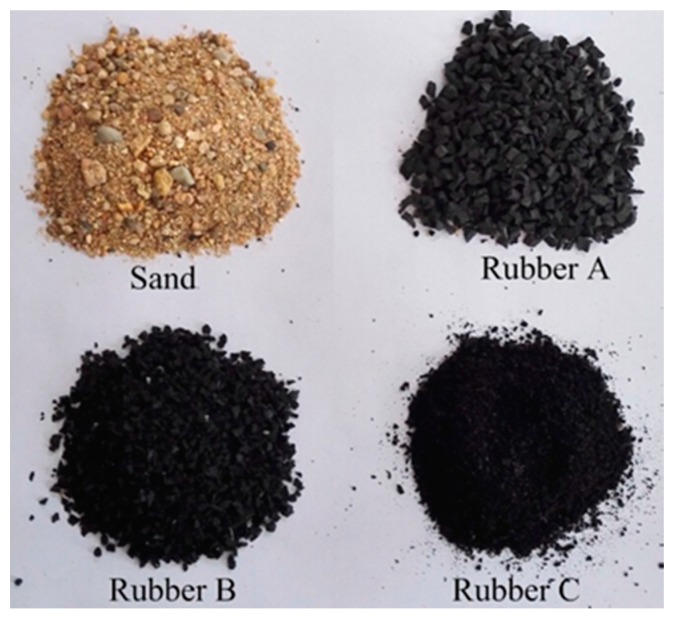
Fine aggregates: Sand, Rubber A, Rubber B, and Rubber C.

**Figure 2:**
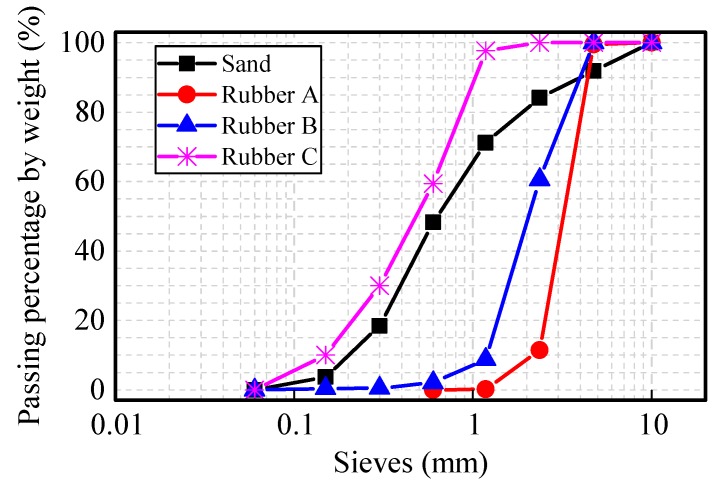
Particle size distribution of fine aggregates.

P.O42.5 grade ordinary Portland cement (compressive strength is 47.7 MPa at the age of 28 days by the test method of Chinese standard GB/T 17671-1999) produced in Tianjin was used in the study. The chemical composition of the cement is shown in Table 1, given by the supplier. The fine aggregates included sand and three groups of rubbers, as shown in Figure 1. The sand used was local river sand. All the rubber aggregates were obtained from the mechanical grinding of used tires. Three groups of rubber crumbs were selected in this study. They were named Rubber A (major particle size 2–4 mm), Rubber B (major particle size 1–3 mm), and Rubber C (major particle size 0–2 mm). Size analyses of aggregates were performed using the sieve method. The chemical ingredients of crumb rubber are listed in Table 2. The particle size distribution of fine aggregates is shown in Figure 2.

The mixture proportion of M0 was taken as the control group, with a water-to-cement ratios (W/C) of 0.5 and a cement-to-sand (C/S) ratio of 0.4. The density of sand was 2650 kg/m^3^. The density of the three kinds of rubbers was set to 1060 kg/m^3^, which was the average density of Rubber A, Rubber B, and Rubber C. Rubber particles were introduced in the mortar mixture by the partial volume substitution of sand. The mix proportions of rubberized mortars are presented in Table 3. Mortar mixes were designated considering the rubber particle size and mixing proportions allowing them to be referenced easily. For example, the mix M-17%-RA, refers to 17% rubber cement mass ratio and rubber aggregate A.

### 2.2. Specimen Preparation and Test Methods

**Figure 3:**
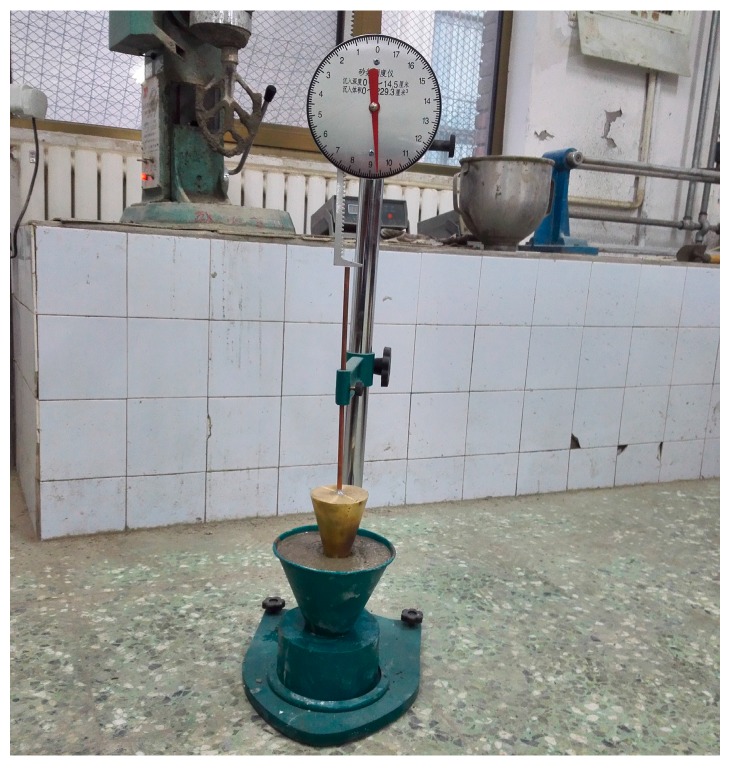
Consistency test of mortars.

The consistency and density of fresh mortar were tested according to JGJ/T70-2009 [31]. The consistency test, as shown in Figure 3, was conducted as follows. First, we wiped the mortar container and the surface of slump cone with wet cloth, and poured mortar mixture into the container. Second, we tamped the mortar 25 times with a tamping rod, and maintained the sharp point of the slump cone at the middle of the mortar surface. Third, the slump cone was allowed to fall freely, and its falling depth was recorded as the consistency value of the mortar. Test results for both the consistency and density of mortars were the average of three measured values.

The pore size distribution was determined via the mercury intrusion porosimetry (MIP) method, which is a typical means of measuring the pore size distribution in cement-based materials [32,33,34], by using an AutoPoreIV9500, which can measure pore sizes not less than 6 nm. The mortar specimens were crushed and placed in ethanol solution to avoid hydration. Small pieces of mortar, around 10 mm, were taken from the middle of the mortar specimen by a hand clipper. The samples for the MIP test (which requires the total removal of moisture) were dried in an oven at 105 °C for 4 h.

In total 30 specimens with dimensions of 40 mm × 40 mm × 160 mm were prepared for compressive strength tests, and, after flexural strength tests, 60 specimens were tested for compressive strength in accordance with GB/T 17671-1999 [35]. Thirty specimens with dimensions of 100 mm × 100 mm × 100 mm were prepared for the splitting tensile strength test. In total 60 specimens with dimensions of 70.7 mm × 70.7 mm × 210 mm were prepared for the elastic modulus tests, 30 specimens for axial compressive strength test and 30 specimens for elastic modulus in compression test, according to Chinese standard JGJ70-1990 [36]. All specimens were removed from the molds after one day, and then standard cured. The compressive strength, flexural strength, splitting tensile strength, and elastic modulus of the specimens were measured on the 28th day. The average of three measured values was taken as the test result in each of the tests conducted.

**Figure 4:**
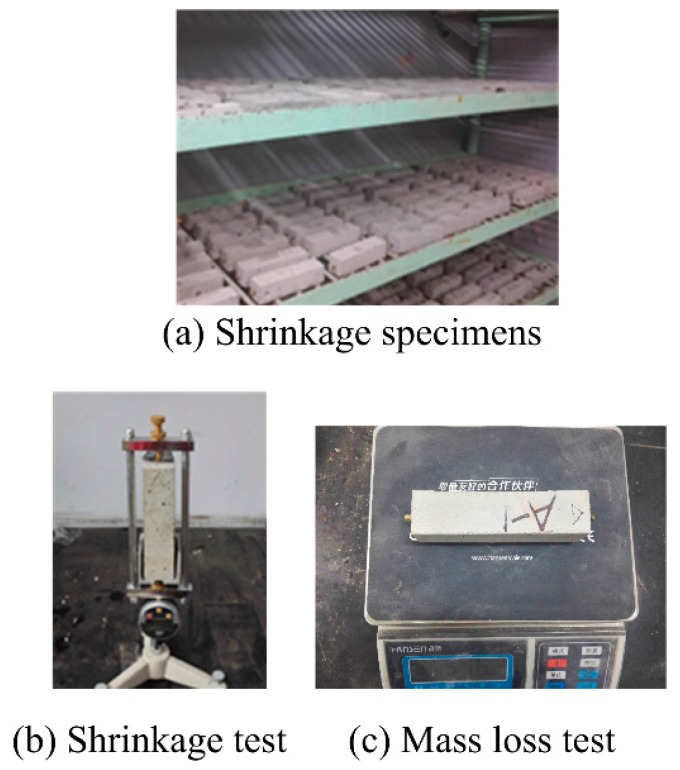
Shrinkage test of mortars.

Mortar was cast in a rigid steel mold to prepare the specimens for shrinkage test. The dimensions of the mortar specimens were 40 mm × 40 mm × 160 mm. Two copper studs were embedded at the center of both ends of the specimen. The specimens were brought into the curing room after pouring. The specimens were removed from the mold after seven days. The drying shrinkage of CRM is conducted according to Chinese standard JGJ/T70-2009 [31]. The initial weight and length of the specimens were measured immediately. Afterward, the specimens were cured in a natural drying curing room at a temperature of 20 ± 2 °C and relative humidity (RH) of 50% ± 5%, as shown in Figure 4a. The length change and mass loss of the specimens, as shown in Figure 4b,c, were measured on days 2, 7, 14, 21, 28, 35, 42, 84, 98, 119, and 140.

## 3. Results and Discussion

### 3.1. Properties of Fresh Mortars

**Figure 5:**
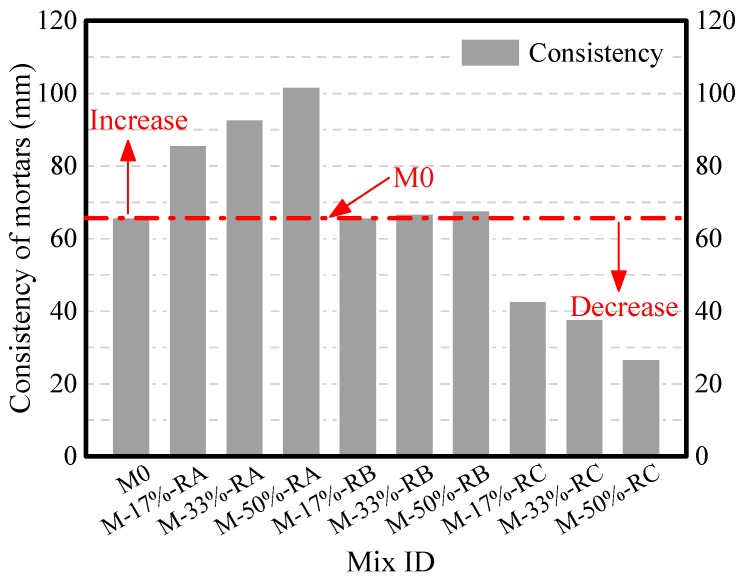
Consistency of crumb rubber mortars.

Figure 5 shows the consistency of mortars with rubbers of different sizes. The consistency of the mortar is found to be influenced by the size and content of rubber used. The consistency of the mortar mixed with Rubber A increased compared with that of the plain one, whereas the consistency of the mortar mixed with Rubber C decreased compared with that of the plain one. With the rubber cement ratio of 50%, the consistency of the mortars mixed with Rubber A and Rubber B increased by 54.5% and 0.3%, whereas the consistency of the mortars mixed with Rubber C decreased by 59.1% compared with that of plain mortar M0. The consistency of mortar decreases with a decrease in the rubber size because larger surface areas were produced. With larger surface areas, more cement paste is needed for covering the surface of rubbers, so the thickness of the cover layer decreases with the increase of rubber surface area. The decrease of cover layer thickness leads to the reduction of cement paste lubrication, which eventually leads to the reduction of mortar consistency.

**Figure 6:**
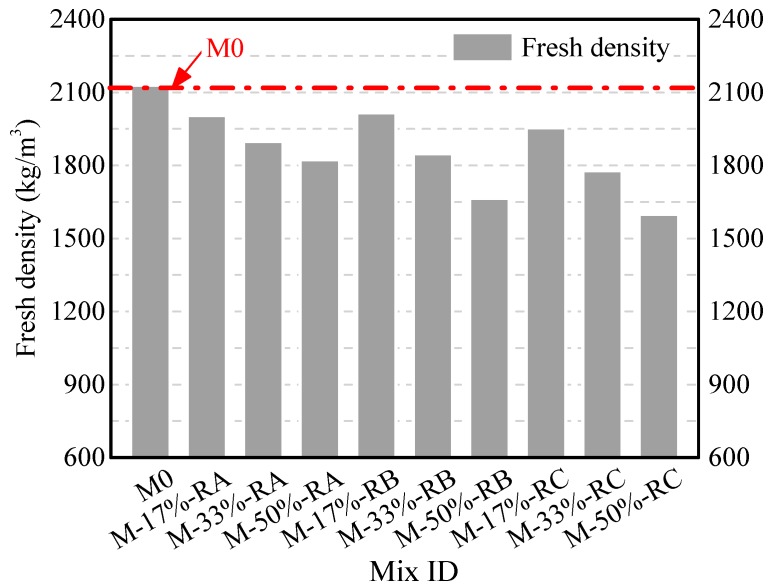
Fresh density of crumb rubber mortars.

Figure 6 shows the fresh density of mortars with rubber of different sizes. The fresh density of mortar was found to decrease with the increase of the rubber content. The effects of crumb rubber on density were more pronounced when smaller sized crumb rubber was used. At the same rubber cement mass ratio, the mortar mixed with Rubber C exhibited lower density than the one mixed with Rubber B or Rubber A. With the same rubber cement mass ratio of 50%, the fresh densities of mortars mixed with Rubbers A, B, and C were reduced to 14.4%, 21.8%, and 24.9%, respectively, compared with those of the plain mortar.

### 3.2. Porosity Structures of Hardened Rubberized Mortars

**Figure 7:**
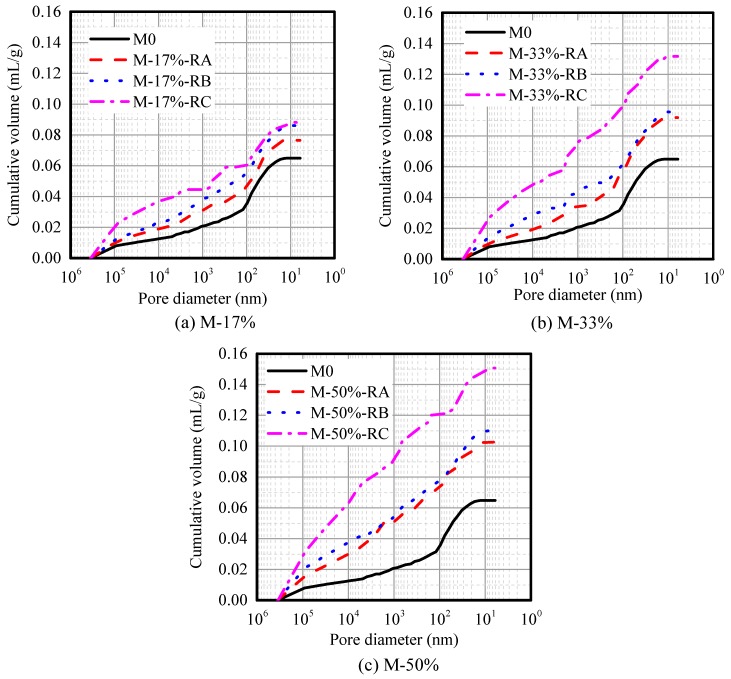
Pore distribution for CRMs.

Figure 7 shows the pore size distribution for rubberized mortars with different rubber sizes and contents. Table 4 presents the total pore and mesopore (<50 nm) volumes of CRMs. Figure 7 shows that the cumulative volume increases with the rubber content or decrease of the rubber size, and the influences of Rubber C on the pore volume of CRM is very significant. With the smallest size and largest content of rubber, the cumulative porosity volume of sample M-50%-RC is the largest, which is 2.32 times that of the plain mortar M0. Mesopore is a pore <50 nm, which is directly related to the drying shrinkage. Table 4 shows that the mesopore volume also increases with an increase in the rubber content. However, in terms of rubber size, especially at rubber cement ratios of 17% and 33%, the mesopore volumes of CRM showed slight difference. On the basis of the above analyses, we conclude that both the rubber content and size influence the pore volume, but the mesopore volume is mostly affected by the rubber content.

### 3.3. Mechanical Properties of CRM

Table 5 shows that the compressive strength, flexural strength, splitting strength, and elastic modulus of rubberized mortar demonstrate a decreasing tendency with the decreasing of rubber size and the increasing mixing ratio of rubber particles content. The reduction effects of rubber on cement mortars are obvious, especially with a large quantity of rubber mixing. With the same content of rubber, the reduction effect is more significant when a smaller size of rubber is used, which is the same as the results of paper [37]. The largest reduction of strength occurred when the rubber cement ratio was 50% with Rubber C. The compressive strength, flexural strength, splitting strength, and elastic modulus reductions were 77.8%, 57.7%, 70.7%, and 52.8%, respectively, compared with those of M0 on the 28th day.

Generally, a strong relationship exists between the mechanical strength and the porosity of the specimens [38,39]. The influences of rubber on CRM strength reduction are of two aspects. First, the stiffness of rubber is less than that of sand for the elastic modulus of the cement paste to be almost 3000 times that of rubber. Second, as shown in Table 3, more porosity is generated in mortars as the rubber content increases, especially when small sized rubber is used. The two aspects are both attributed to the reduction of CRM mechanical strengths. Smaller sized rubber can lead to more porosity with the same rubber content. Therefore, the mechanical strength of CRM decreases as the size of rubber decreases.

### 3.4. Drying Shrinkage and Mass Loss

#### 3.4.1. Drying Shrinkage Results

**Figure 8:**
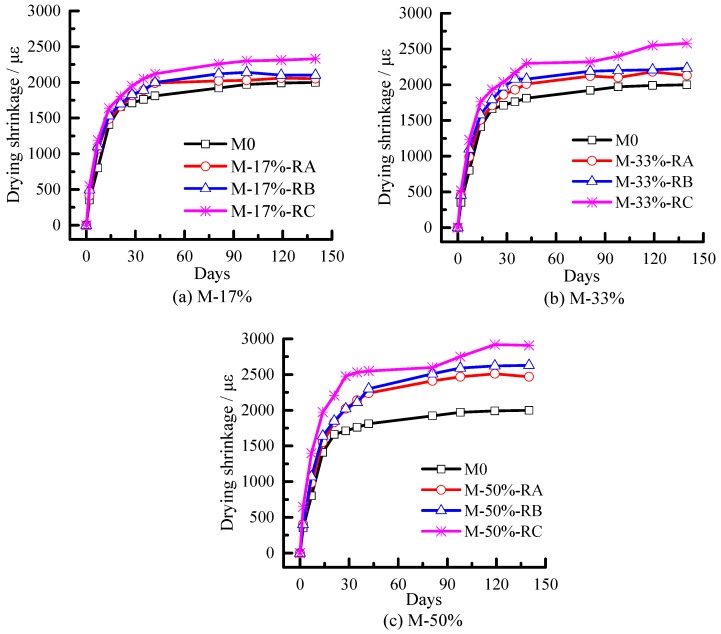
Drying shrinkage of mortars.

Figure 8 shows the results of the drying shrinkage tests on the rubberized mortars. The drying shrinkage of the rubberized mortars appeared to depend on the size as well as crumb rubber content. The shrinkage of mortars increased with the rubber contents, whereas mortars containing smaller rubber particles appeared to shrink much more than those with large rubber particles. The maximum shrinkage strains were observed in mortars with the highest rubber cement ratio of 0.5 and smallest particle size (Rubber C), which is 1.46 times that of the shrinkage value of plain mortar (M0) on the 140th day.

#### 3.4.2. Mass Loss Results

**Figure 9:**
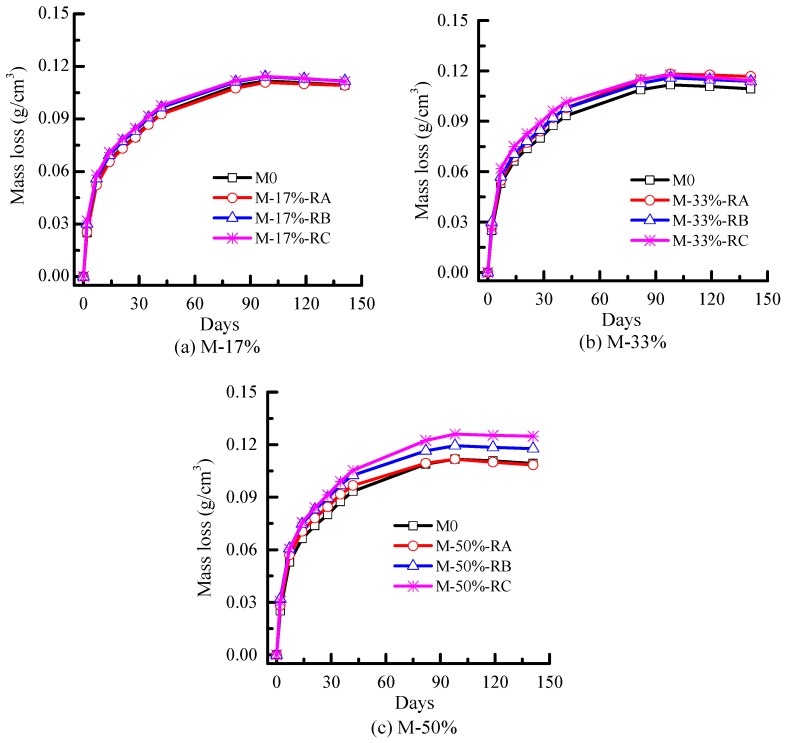
Mass loss of mortars.

The mass loss caused by water evaporation from mortar was measured at R.H. 50% ± 5% and 20 °C, as shown in Figure 9. The amount of water evaporation mainly followed the rubber content increase trends. However, the influences of rubber content and size on mass loss were not as remarkable as their effects on drying shrinkage. For example, both the mass loss and shrinkage of M-50%-RC are the largest of the 10 mixes, and the increase proportion of mass loss was 14% larger than M0, whereas the increase proportion of drying shrinkage was 46% larger than M0. Furthermore, although the mass loss decreased slightly after 100 days, the drying shrinkage still increased slightly. The mass loss decrease (mainly from 100 days to 140 days) may be caused by carbonation, which refers to the reaction of the cement hydration product with carbon dioxide on the exterior surface of the cement-based materials exposed to natural environmental conditions.

**Figure 10:**
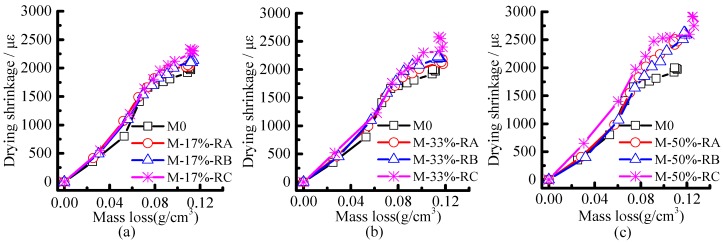
Relationship between mass loss and drying shrinkage of rubberized mortars, (**a**) M-17%; (**b**) M-33%; (**c**) M-50%.

The drying shrinkage of mortar is plotted versus the mass loss in Figure 10a–c. The drying shrinkage increased with the water evaporation until a certain age (about day 100), as expected. Figure 10a–c show that with the same mass loss the drying shrinkage of mortars with Rubber C is the largest, followed by mortars with Rubber B and Rubber A.

#### 3.4.3. Discussion

Drying shrinkage is usually referred to as the main consequence of capillary tension and disjoining pressure [40]. Drying shrinkage is known to be mainly caused by capillary tension caused by the loss of water from the capillary pores [41]. As an example of a capillary pressure of an ideal pore, liquid tension may be quantified by the Laplace equation (Equation (1)):

(1) $$\sigma =2\gamma /r_{s}$$

where σ (Pa) is the capillary pressure of a cylindrical ideal pore, g (N/m) is the surface tension of the pore fluid, and $r_{s}$ (m) is the porosity radius. The higher capillary force, which results from mesopore (<50 nm), will increase the dry shrinkage [33,41,42]. There are two main factors: capillary porosity content and the ability to resist deformation, which affected the drying shrinkage of mortars. First, mortars with high capillary porosity content shrink more; second, mortars with small ability to resist deformation shrink more.

As discussed above, mesopore porosity, which is related to capillary tension, mostly increases with the increase of rubber content, whereas total pore volume, which is related to the ability to resist deformation, mostly increases with the increase of rubber content or decrease of rubber size. Because rubber size has little influence on capillary tension and mainly influences the ability to resist deformation of CRM, it can be concluded that the rubber size influences the drying shrinkage mainly through its influence on the ability to resist deformation reduction of CRM.

## 4. Conclusions

This research mainly discussed the influence of rubber size on the consistency, fresh density, pore structures, mechanical properties, and drying shrinkage properties of CRMs. Rubber content and size can affect the porosity structures of CRM, and the reasons for the strength reduction and drying shrinkage increase of CRM are the combined changes in rubber content and porosity structures. The conclusions can be summarized as follows:

The consistency of CRM decreases as the size of rubber decreases. The consistencies of CRM with Rubber A, Rubber B, and Rubber C are respectively larger than, similar to, and smaller than M0. The fresh density of CRM with the same rubber content decreases with a decrease in rubber size, because the total pore volume of CRM of small rubber size is larger.

Both the rubber content increase and rubber size decrease contribute to the increase in the total pore volume. However, the rubber size does not significantly influence the mesopore volume, and the mesopore volume of CRM increases mostly because of rubber content increase.

The mechanical properties of CRM decrease as the rubber content increases or the size of rubber decreases. The low stiffness of rubber and large pore volumes, attributed especially to rubber of small size, both contribute to the reduction of CRM strength.

The drying shrinkage of CRM increases as the rubber content increases or size decreases. With the same mass loss and rubber content, the drying shrinkage of CRM with Rubber C is the largest among the three. However, the rubber size does not significantly influence the mesopore volume. Therefore, the rubber size influences the drying shrinkage mainly through its reduction effects on the ability to resist the deformation of CRM.

## Figures and Tables

**Table 1:** Chemical composition of P.O42.5 grade ordinary Portland cement.

Chemical Compound	CaO	SiO_2_	Al_2_O_3_	Fe_2_O_3_	SO_3_	MgO	Lgnition Loss
Percentage (%)	63.11	22.60	5.03	4.38	2.24	1.46	1.18

**Table 2:** Chemical ingredients of crumb rubber (mass fraction %).

Rubber Hydrocarbon	Carbon Black	Acetone Extract	Isoprene	Water	Ash Content	Fiber Content	Metal Content	Others
45.2	25.8	14.2	12.1	0.8	0.9	0.5	0.08	0.42

**Table 3:** Mortar mixture proportions (by weight).

Mix	Water	Cement	Sand	Rubber (A/B/C)
M0	0.50	1	2.50	0
M-17%	0.50	1	2.08	0.17
M-33%	0.50	1	1.68	0.33
M-50%	0.50	1	1.25	0.50

**Table 4:** Total pore volume and capillary pore volume of CRMs.

Sample	Pore Volume (mL/g)	
	Total Pore Volume	Mesopore (<50 nm) Volume
M0	0.0650	0.0139
M-17%-RA	0.0765	0.0168
M-33%-RA	0.0919	0.0186
M-50%-RA	0.1026	0.0191
M-17%-RB	0.0861	0.0171
M-33%-RB	0.0956	0.0196
M-50%-RB	0.110	0.0224
M-17%-RC	0.0881	0.0151
M-33%-RC	0.1316	0.0200
M-50%-RC	0.1506	0.0267

**Table 5:** Test results of compressive strength, flexural strength, splitting strength, and elastic modulus.

Sample	Compressive Strength/MPa	Flexural Strength/MPa	Splitting Strength/MPa	Elastic Modulus/GPa
M0	49.2	7.4	3.7	25.2
M-17%-RA	35.7	6.3	2.7	21.2
M-33%-RA	27.4	5.3	1.9	17.5
M-50%-RA	11.7	3.5	1.5	14.8
M-17%-RB	37.2	6.1	2.2	20.3
M-33%-RB	24.3	4.9	1.7	16.0
M-50%-RB	12.5	3.3	1.3	13.1
M-17%-RC	28.5	5.6	2.0	18.2
M-33%-RC	19.4	4.3	1.4	14.9
M-50%-RC	10.9	3.1	1.1	11.9
